# Cone‐Rod Dystrophy and Progressive Visual Loss as the First Manifestation of Neuronal Ceroid Lipofuscinosis Type 7: A Case Report

**DOI:** 10.1002/ccr3.9566

**Published:** 2024-11-15

**Authors:** Seyedeh Maryam Hosseini, Reza Nejad Shahrokh Abadi, Meisam Babaei, Fatemeh Eghbal, Narges Hashemi

**Affiliations:** ^1^ Eye Research Center Mashhad University of Medical Sciences Mashhad Iran; ^2^ Faculty of Medicine Mashhad University of Medical Sciences Mashhad Iran; ^3^ Department of Pediatrics North Khorasan University of Medical Sciences Bojnurd Iran; ^4^ Next Generation Genetic Polyclinic Mashhad Iran; ^5^ Department of Pediatrics School of Medicine Mashhad University of Medical Sciences Mashhad Iran

**Keywords:** cone‐rod dystrophy, NCL, neurodegeneration, pediatric ophthalmology, retinal degeneration

## Abstract

This case report documents the experience of a 5‐year‐old girl who showed signs of retinal degeneration as the initial symptom of neuronal ceroid lipofuscinosis (NCLs). She originally presented with visual failure, which rapidly progressed to near total bilateral blindness. Two years later, she developed seizures and cognitive impairment, leading to a diagnosis of NCL7 resulting from a homozygote mutation in the MFSD8 gene. This case underscores the importance of considering NCLs as a potential diagnosis in cases of cone‐rod dystrophy and visual loss as the primary clinical feature. It also emphasizes the early onset and initial presentation of retinal degeneration associated with NCL7, before other signs and symptoms manifest, as the second documented case of its kind. Due to the potential for NCL7 to present initially with visual loss before other hallmark signs, it is crucial to consider it among various syndromic and non‐syndromic disorders in the differential diagnosis.

**Summary**

- Neuronal ceroid lipofuscinosis type 7 is a rare form of neurodegeneration caused by abnormalities in lysosomal storage.
- This disorder is typically characterized as having central nervous system manifestation including motor deterioration, seizures, and visual loss.
- However, rarely this visual loss can be as a result of changes within the retina as opposed to involvement of the visual pathway in the brain.
- Rarely, these changes can involve the dystrophy of the cone and rod photoreceptors.

## 1 Introduction

Neuronal ceroid lipofuscinosis (NCLs), often known as Batten disease, are a series of hereditary neurodegenerative illnesses caused by lysosomal storage abnormalities [1]. These disorders affect both sexes and have an incidence of approximately one in every 100,000 live births [2]. Currently, 13 subtypes have been identified and classified based on their different mutagenic origin, onset age, primary symptoms, and illness course progression, with the typical age of onset being in the first decade [1]. Pediatric NCL is frequently characterized by a combination of vision loss, dementia, seizures, and motor deterioration, with few extra‐CNS symptoms [1]. Although gradual vision loss is a common symptom among NCLs, few documented examples have been described in which visual impairment is the initial clinical sign. Brain MRI of NCL patients reveals diverse findings, including hypointensities in the Thalami, hyperintensities in the periventricular white matter, and cerebral or cerebellar atrophy [3]. Electrodiagnostic procedures, such as electroretinography (ERG), show diminished retinogram amplitudes in some cases, while optical coherence tomography (OCT) can display atrophy and cone‐rod dystrophy, with genetic testing being routinely required to confirm the diagnosis [4]. In this case report, we detail the clinical and paraclinical findings of a child presenting initially with prolonged visual failure as a result of NCL7 mutation, as the first documented report of its kind.

## 2 Case History/Examination

The patient is a 5‐year‐old girl born to asymptomatic and consanguineous parents. Her prenatal and perinatal history were unremarkable, and she had normal physical and intellectual development during infancy. At the age of 2, she began to experience a gradual decline in vision, which progressed to near total bilateral blindness by the age of 5. Fundus examination revealed diffuse retinal degeneration with prominent macular atrophy consistent with cone‐rod dystrophy.

## 3 Differential Diagnosis, Investigations, and Treatment

**FIGURE 1:**
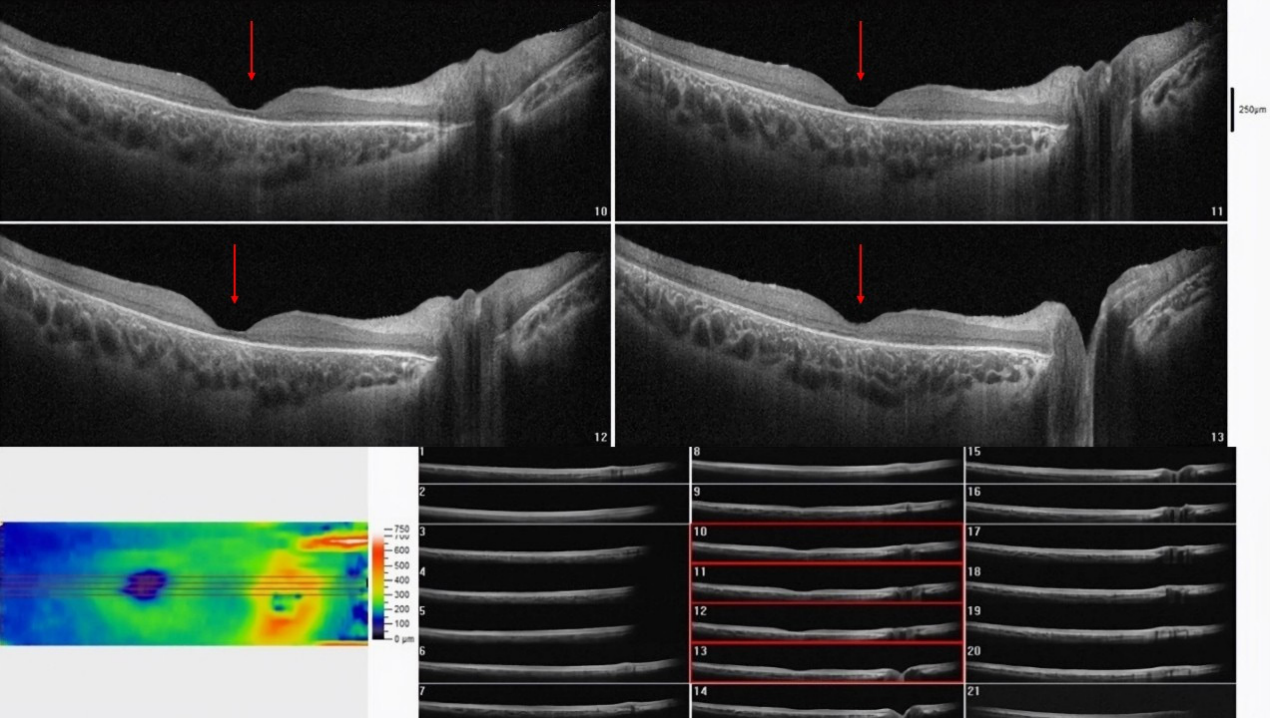
Macular optical coherence tomography (OCT) of the right eye showed significant foveal atrophy and outer retinal layer thinning, indicating photoreceptor destruction resembling cone dystrophy (red arrows).

**FIGURE 2:**
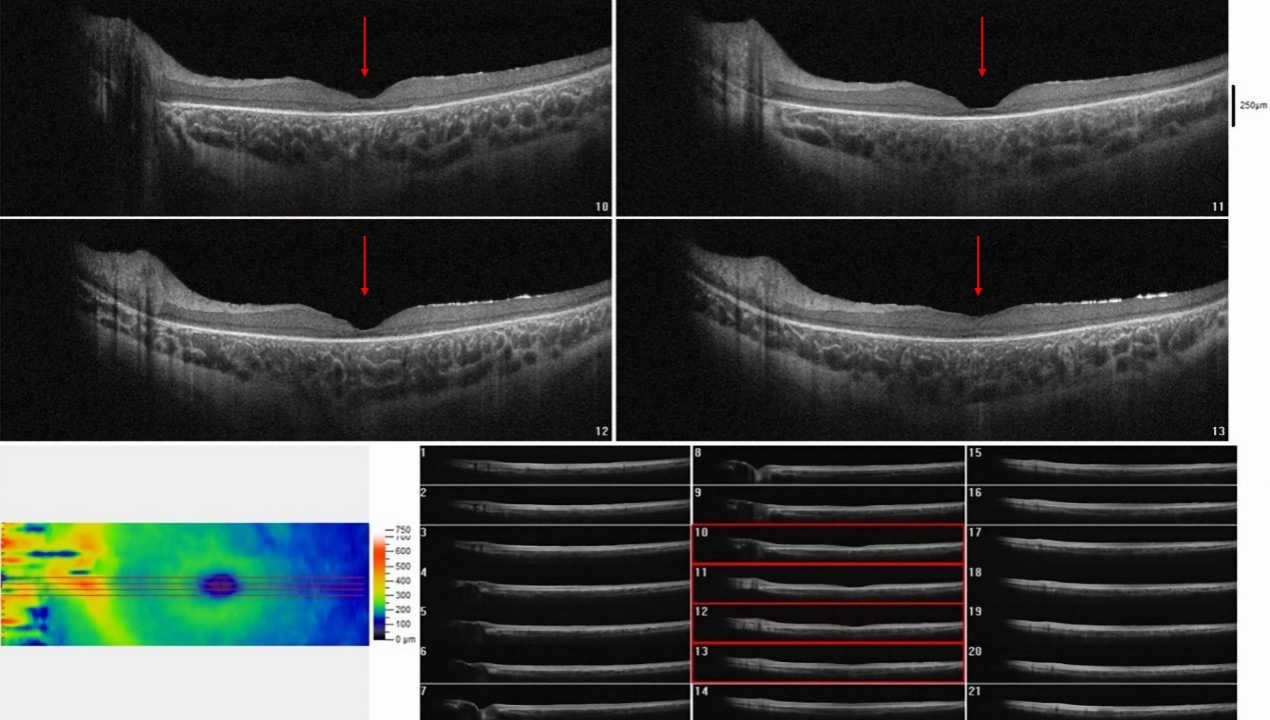
Macular optical coherence tomography (OCT) of the left eye also showed significant foveal atrophy and outer retinal layer thinning (red arrows).

A previous OCT performed showed changes consistent with cone‐rod dystrophy, including diffuse retinal thinning, particularly at the center of the macula (foveal atrophy) (Figures 1 and 2). Significant destruction of the outer retinal layer and attenuation of photoreceptors were observed, however fundoscopy revealed normal optic discs in both eyes. Six months prior to the visit, at the age of 4 and a half years, the patient started experiencing epileptic seizures at a rate of one seizure per month, characterized by cyanosis and lateral gaze. Furthermore, the parents observed gradual cognitive impairment and psychosocial regression over the past year, which was affirmed by a routine neurologic examination on admission. Consequently, a brain MRI was conducted, revealing nonspecific and mild widespread cerebral atrophy, as well as periventricular hyperintense signals. Whole exome sequencing (WES) was subsequently carried out to confirm the suspected diagnosis of a metabolic disorder. Bioinformatic analysis revealed a homozygote c.616C>T mutation in MFSD8 (NM_152778.2) which leads to replacement of the stop codon instead of glutamine (p.Gln206*). According to the ACMG guideline using online bioinformatic tools along with clinical manifestation, it was classified as a likely pathological variant associated with neuronal ceroid lipofuscinosis type 7 (OMIM:610951).

## 4 Conclusion and Results (Outcome and Follow‐Up)

As of now the patient is nearly blind in both eyes, with visual acuity of only determining finger counts and hand motion. The patient received a final diagnosis of NCL7, as the cause of her seizures, cognitive impairment, and cone‐rod dystrophy. She was instructed to routinely follow‐up with an ophthalmologist to assess changes in vision, and to undergo yearly brain MRIs to document any alterations.

## 5 Discussion

Each variant of NCL exhibits a distinct disease progression and onset age, often differentiated by the patient's medical history, which may offer clues regarding the specific genetic mutation. NCL type 7 typically begins in late infantile, with most cases occurring between the ages of 2 and 8; however, genetic diagnosis generally occurs months after the initial symptoms appear [5]. The most common early symptoms include seizures followed by myoclonus, with subsequent manifestations of psychosocial and developmental regression, as well as cognitive decline [5]. Visual impairment may also develop as the disease progresses, although this is considered rare as an initial symptom, with only one prior documentation of this manifestation [6]. This type of initial presentation is more commonly noted among NCL3 patients, however, as noted by Kousi et al., NCL7 is considered as a differential diagnosis if the patient tests negative for NCL3 [7]. In this particular case, the patient initially experienced a gradual loss of vision in both eyes, followed by seizures, regression, and cognitive impairment 2 years later. NCL7 is associated with retinal degeneration, presenting as macular or cone‐rod dystrophy [5], with OCT imaging of NCL7 patients presenting with visual impairment typically revealing atrophy of the outer retina in the macula, along with thinning of the fovea and diffuse central and parafoveal photoreceptor loss [4]. Due to the initial presenting symptom in this case being visual impairment along with macular atrophy on OCT, in addition to the patient being devoid of systemic manifestations, an initial diagnosis of cone‐rod dystrophy was made. However, as the symptoms progressed and the patient deteriorated neurologically, this diagnosis was put into question. Cerebral atrophy is a prominent radiological feature in NCL subtypes, particularly in late‐juvenile variants [3]. Additionally, white matter signal abnormalities, such as increased signaling in the periventricular areas, are commonly observed on T2‐weighted images [3]. Both of these findings were found in this patient, although some typical NCL type 7 MRI findings such as predominant cerebellar‐over‐cerebral atrophy along with T2/FLAIR hypointense thalami were not noted [8]. Because of the irregular and atypical nature of these findings, as well as the apparent syndromic nature of the condition, Whole Exome Sequencing was performed. This revealed a homozygous mutation in the MFSD8 gene, ultimately leading to the diagnosis of NCL type 7.

In essence, NCLs encompass a group of inherited lysosomal storage disorders, with NCL type 7 representing a rare subtype caused by mutations in the MFSD8 gene. The accumulation of ceroid and lipofuscin within the lysosome results in neuronal death, leading to a combination of symptoms including seizures, cognitive decline, psychosocial regression, and visual loss. While all these symptoms were present in this patient, the occurrence of visual loss as the primary symptom, preceding the onset of seizures and cognitive decline by years, is particularly noteworthy. This case report represents the second documented instance of this occurrence. The initial diagnosis of cone‐rod dystrophy was called into question only when additional symptoms emerged and an MRI was conducted. Therefore, it is crucial to consider NCL7 when diagnosing cone‐rod dystrophy, even in the absence of initial neurological deficits.

## Author Contributions

**Seyedeh Maryam Hosseini:** conceptualization, methodology, validation. **Reza Nejad Shahrokh Abadi:** investigation, writing – original draft, writing – review and editing. **Meisam Babaei:** data curation, investigation, supervision, validation. **Fatemeh Eghbal:** conceptualization, formal analysis, project administration, resources, writing – review and editing. **Narges Hashemi:** conceptualization, data curation, investigation, methodology, supervision, validation, writing – review and editing.

## Consent

Written informed consent was obtained from the patient to publish this report in accordance with the journal's patient consent policy.

## Conflicts of Interest

The authors declare no conflicts of interest.

## Data Availability

Data supporting the conclusions of the study is all available free of cost through open access journals and websites.
